# Steps to achieve quantitative measurements of microRNA using two step droplet digital PCR

**DOI:** 10.1371/journal.pone.0188085

**Published:** 2017-11-16

**Authors:** Erica V. Stein, David L. Duewer, Natalia Farkas, Erica L. Romsos, Lili Wang, Kenneth D. Cole

**Affiliations:** 1 Biosystems and Biomaterials Division, Materials Measurement Laboratory, National Institute of Standards and Technology, Gaithersburg, Maryland, United States of America; 2 Chemical Sciences Division, Materials Measurement Laboratory, National Institute of Standards and Technology, Gaithersburg, Maryland, United States of America; 3 Engineering Physics Division, Physical Measurement Laboratory, National Institute of Standards and Technology, Gaithersburg, Maryland, United States of America; 4 Biomolecular Measurement Division, Materials Measurement Laboratory, National Institute of Standards and Technology, Gaithersburg, Maryland, United States of America; University of Connecticut Health Center, UNITED STATES

## Abstract

Droplet digital PCR (ddPCR) is being advocated as a reference method to measure rare genomic targets. It has consistently been proven to be more sensitive and direct at discerning copy numbers of DNA than other quantitative methods. However, one of the largest obstacles to measuring microRNA (miRNA) using ddPCR is that reverse transcription efficiency depends upon the target, meaning small RNA nucleotide composition directly effects primer specificity in a manner that prevents traditional quantitation optimization strategies. Additionally, the use of reagents that are optimized for miRNA measurements using quantitative real-time PCR (qRT-PCR) appear to either cause false positive or false negative detection of certain targets when used with traditional ddPCR quantification methods. False readings are often related to using inadequate enzymes, primers and probes. Given that two-step miRNA quantification using ddPCR relies solely on reverse transcription and uses proprietary reagents previously optimized only for qRT-PCR, these barriers are substantial. Therefore, here we outline essential controls, optimization techniques, and an efficacy model to improve the quality of ddPCR miRNA measurements. We have applied two-step principles used for miRNA qRT-PCR measurements and leveraged the use of synthetic miRNA targets to evaluate ddPCR following cDNA synthesis with four different commercial kits. We have identified inefficiencies and limitations as well as proposed ways to circumvent identified obstacles. Lastly, we show that we can apply these criteria to a model system to confidently quantify miRNA copy number. Our measurement technique is a novel way to quantify specific miRNA copy number in a single sample, without using standard curves for individual experiments. Our methodology can be used for validation and control measurements, as well as a diagnostic technique that allows scientists, technicians, clinicians, and regulators to base miRNA measures on a single unit of measurement rather than a ratio of values.

## Introduction

MicroRNAs (miRNA) are short noncoding RNA oligonucleotides that were discovered in *Caenorhabditis elegans* over two-decades ago. Upon their discovery, miRNAs were thought of as mundane epigenetic regulators of gene expression. Since that time, researchers have uncovered notable roles for miRNA in almost every area of biology including cell-to-cell communication, gene regulation, metabolism, and host-pathogen response [1]. Their ubiquitous functions have made them direct targets for diagnostic, prognostic, and therapeutic discovery, however their approval for clinical use has encountered many regulatory and practical obstacles.

miRNAs are notoriously difficult to measure using conventional clinical techniques such as standard or quantitative real-time polymerase chain reaction (qRT-PCR) or microarray [2]. Principles leveraged for years to optimize DNA- or mRNA-based qRT-PCR assays and microarrays often cannot be used similarly for miRNA measurements. For example, endogenous controls are necessary in addition to standard curves to calculate exact copy number using qRT-PCR. However, there are no stable, ubiquitous endogenous controls that can be used for normalization when quantifying miRNA [3]. miRNA levels can be below detection limits of conventional qRT-PCR or fold change can be too discrete for microarray detection [4]. Whereas some investigators have relied on pre-amplification to circumvent this challenge, reviews are mixed on whether this skews the data disproportionally, especially among certain targets [5, 6]. And while microarray and qRT-PCR measurement technologies have been shown to be a valid method for determining fold differences between different samples [4], a widely recognized challenge is how to measure individual miRNA copy or concentration at limiting dilutions accurately and without bias [7, 8]. Digital PCR (dPCR) has shown significant potential as a new measurement capability to solve many of these specific issues.

The measurement community has often tried to overcome metrology challenges by expanding technological capability and breath. Digital PCR uses real-time or end-point data collection to separate targets into partitions. These partitions are either made by separating copies into physical chambers via microfluidics or by creating water-in-oil immersion droplets that hydrostatically separate targets. Respective technologies are named chamber dPCR (cdPCR) and droplet dPCR (ddPCR). Given a few basic quantitative assumptions, such as (1) targets independently segregate, (2) targets are fully accessible to probes and primers, (3) droplet volume is known, and (4) each partition contains a limited number of targets, then absolute copy number of genomic material can be calculated by applying Poisson correction [9, 10].

One caveat of using dPCR for miRNA measurements is that some of the original limitations still exist, which creates the same measurement uncertainty seen with qRT-PCR or microarray. For example, miRNA has such short target sequences that primer and probe optimization strategies, such as melting temperature, length, and guanine-cytosine content, are inherently more difficult and off-target hybridization with heterogeneous samples or low yield via molecular dropout can occur [9, 11]. Additionally, dPCR does not seem to rectify problems associated with performing either one-step or two-step PCR reactions. One-step means that reagents for both cDNA synthesis and PCR are combined in one mix or container and two-step means that cDNA synthesis is independent of PCR [12]. In both one-step and two-step reactions, miRNA must be reverse transcribed to cDNA before PCR can be performed. For our studies we focus on two-step ddPCR, although one-step dPCR, two-step dPCR, one-step qRT-PCR and two-step qRT-PCR are all associated with inherent inefficiencies introduced within the reverse transcription step [6]. Technology to directly and accurately count miRNA is still limited.

Nevertheless, one of the clear benefits to using ddPCR to quantify miRNA is that scientists can avoid some of the perennially unanswered questions like: how do we translate measurement of controlled mixtures to absolute quantification in a single sample [13]? Instead we now enter a new type of debate for this field, one that generally occurs as new and promising technology comes to market. This is the debate over how to use preexisting companion products on new technology. One clear way industry has capitalized on this opportunity is to optimize new proprietary reagents specifically for their instruments. For example, specific ddPCR technologies require certain buffers and enzymes due to oil-droplet compatibility issues and use of homemade or competitor products results in measurement errors [14]. Here our measurement strategies account for all compatibility issues.

Lastly, significant obstacles also exist in the cDNA synthesis step required for two-step ddPCR measurements. Currently, all the miRNA cDNA synthesis kits on the market were created and optimized specifically for qRT-PCR. These products can introduce bias when used with ddPCR reagents [11]. For example, no template controls (NTC) will show amplification of products regardless of the sterility of the experiment [15]. Non-specific positives in template controls, while consistently seen in quantitative ddPCR measurements, have been essentially undetectable using qRT-PCR because of technological limitations, which perhaps lead to the lack of recognition it deserved. However, now that we have accepted these inadequacies we can determine ways to limit and quantify control issues for more exact measurements.

**Fig 1:**
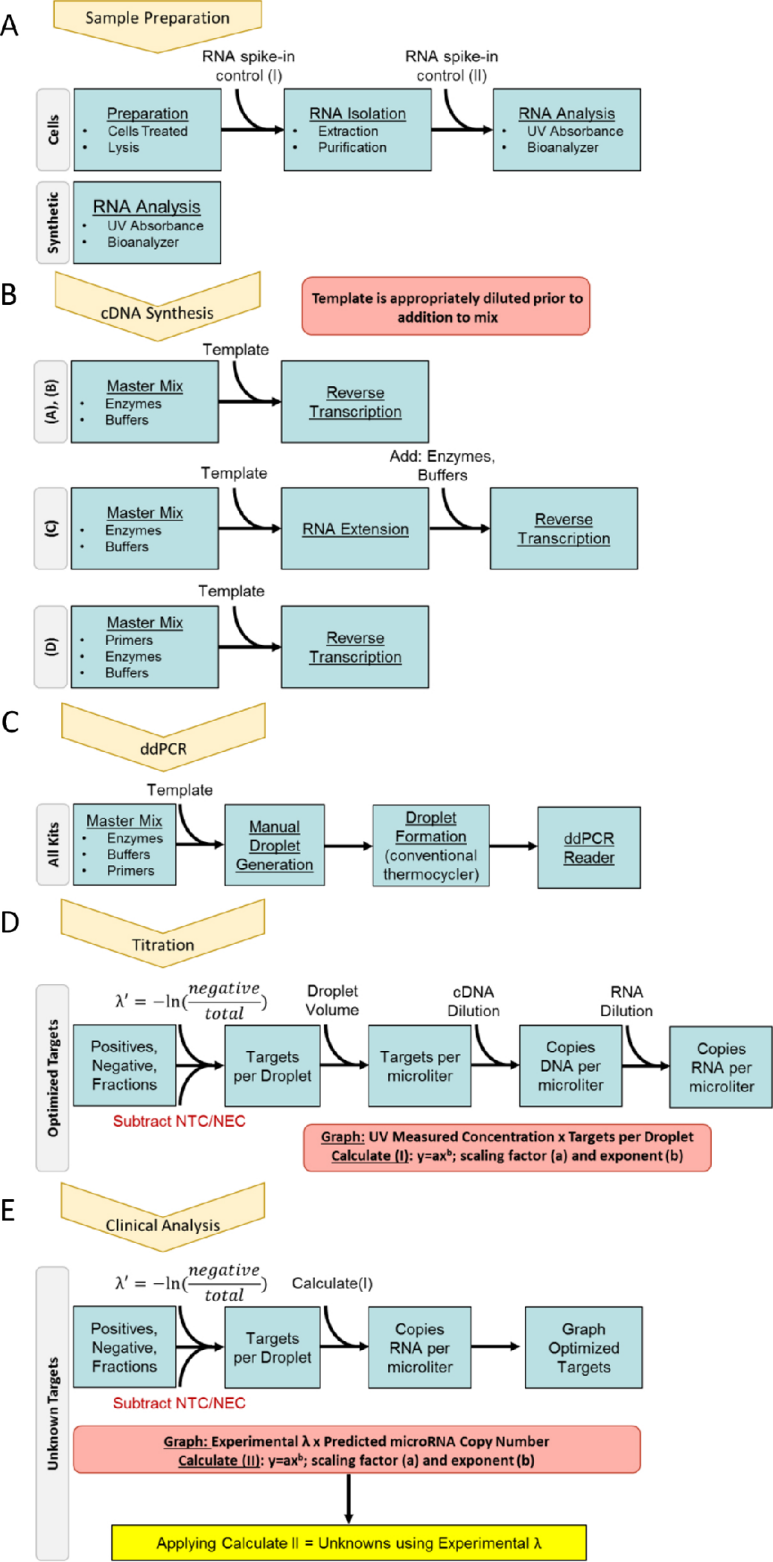
Procedure for analyzing microRNA (miRNA) in a clinical sample. (A) Prepare samples either by isolating total RNA from cells or acquiring synthetic miRNA oligonucleotides. miRNA spike-in controls are added at different points within the RNA extraction process to control for loss of material associated with different steps. (B) cDNA is synthesized using miRNA-specific cDNA synthesis kits. The kits contain reagents that will add adaptor sequences onto the target miRNA so that primers and probes can respectively bind either during cDNA synthesis or during PCR steps. All cDNA synthesis kits show signs of non-specific cDNA products so it is recommended to create one reaction without RNA template (NTC) and one reaction without enzymes (NEC) for each round of cDNA synthesis. (C) Droplet digital PCR (ddPCR) is done by taking the cDNA synthesized from miRNA template and creating water-in-oil droplets that each contain zero to a few target sequences. The cDNA template might need to be diluted to obtain the optimal number of targets per droplet. Results are reported as florescence intensity. Either the user or the instrument software can define a threshold. Droplets that fluoresce at an amplitude higher than the defined threshold will be positive for target sequence and those below threshold are negative for target sequence [16]. (D) Average fraction positive droplet in both the NTC and NEC are subtracted from sample of interest and Poisson distribution can be applied to the remaining fraction negative total droplets. The resultant is the targets per droplet (λ’) and can then be manipulated to give targets per microliter, copies of cDNA per microliter, copies of miRNA per microliter, and concentration. By performing a titration of synthetic miRNA oligonucleotide with a known concentration, one can create a power model that defines how much miRNA input corresponds to a miRNA value following ddPCR. This model will account for any loss assumed in both cDNA synthesis and ddPCR steps. (E) A clinical sample is processed using spike-in controls and validated endogenous miRNA measurements that have been titrated previously. A 96-well plate for a sample contains primers and probes to measure sample-specific values for spike-in controls and validated endogenous. These experimental values are inserted into the power model developed during miRNA titration and converted to predicted values. Graphing experimental targets per droplet (λ’) versus predicted miRNA copies/μL of validated spike-in or endogenous miRNA generates a new power curve that can be applied to all remaining sample targets of interest. These resultants are finite miRNA copy number. Values can be extrapolated to concentration or measurement per cell number using the Avogadro constant or previously measured cell number. Results can be used for clinical diagnosis, prognosis, or treatment purposes.

First, we have designed a few ways to optimize a two-step miRNA dPCR reaction using available cDNA synthesis kits and droplet digital PCR System. Secondly, we evaluated different cDNA Synthesis kits commonly used with these workflows to establish repeatability precision [8], predicted loss, limit of detection, and relative measurement models for targets of interest. We developed protocol recommendations that are agnostic of a specific product so they can be applied evenly to any manufacturer’s product. Lastly, we applied these principles to measure cell-associated miRNA from an established cell line to demonstrate the possibility of using spike-in controls and endogenous markers for more accurate quantification (Fig 1). The use of spike-in controls here is a way to normalize measurements and compute an accurate miRNA copy number. Therefore, using our guidance and recommended work-flow will improve the quality of the miRNA measurements.

## Materials and methods

### Cell culture

THP-1 cell line (TIB-202; ATCC) was purchased directly from American Type Culture Collection for this project (Manassas, VA). In accordance with policy, the research was reviewed and approved by the human subjects review process before research involving human or animal subjects was conducted. THP-1 cells were cultured in RPMI 1640 Medium (Thermo Fisher; Grand Island, NY) containing L-glutamine, penicillin-streptomycin, sodium pyruvate, beta- mercaptoethanol (Sigma; Saint Louis, MO), and endotoxin-free fetal bovine serum (Gemi Bio-Products; Sacramento, CA). THP-1 cells were differentiated by adding 10 ng/mL of phorbol 12- myristate 13- acetate (P8139; Sigma; Saint Louis, MO) to cell culture media without beta- mercaptoethanol (M3148; Sigma; Saint Louis, MO) for 48 h and stimulated with 20 ng/mL ultra-pure Lipopolysaccharide from *E*. *coli* O111:B4 (tlrl-eblps; Invivogen; San Diego, CA) for at least 4 h.

All cells were lifted using Versene/EDTA (Thermo Fisher; Grand Island, NY), flash frozen in liquid nitrogen and stored at -80°C until processing.

### miRNA and total RNA

Mature miRNA sequences were identified using miR-Base (www.mirbase.org, Table 1) [17]. The sequences were used to order synthetic oligonucleotide miRNAs from Integrated DNA Technologies (Coralville, Iowa). Lyophilized synthetic oligonucleotide miRNA was solubilized at the manufacturer’s concentration (Table 1) using RNAase-free 10 mmol/L Tris-HCl 1mM ethylenediaminetetraacetic acid, pH 7.5 buffer solution.

**Table 1:** General information about purchased synthetic oligonucleotides.

miRNA	Sequence^a^	Manufacturer Concentration (ng/μL)^b^	Molecular Weight (g/mol)^b^	Extinction Coefficient L/(mole·cm)^b^
cel-miR-238-3p	UUUGUACUCCGAUGCCAUUCAGA	727.48	6877.4	206800
cel-miR-39-5p	AGCUGAUUUCGUCUUGGUAAUA	698.84	6629.2	202500
hsa-miR-155-5p	UACCCGUAAUCUUCAUAAUCCGAG	739.13	7173.6	222500
hsa-miR-223-3p	UUAAUGCUAAUCGUGAUAGGGGU	685.81	7021.5	202700

^a^Sequence identified using miRbase.

^b^Values supplied by manufacturer.

Total RNA was extracted from cells using a NuceloSpin miRNA kit (Machney-Nagel; Düren, Germany) using manufacturer’s suggested protocol (Fig 1A). Synthetic and cell-associated RNA was stored in individual aliquots at -80°C.

### RNA quantification

Synthetic miRNA oligonucleotides were serially diluted (schematic of dilution shown in S1 Fig) and absorbance was measured (Fig 1A) using a Take3 micro-volume plate on a Synergy MX plate reader (BioTek; Winooski, VT). Data was initially collected using Gen5 software (BioTek; Winooski, VT) and exported into Excel (Microsoft; Redmond, WA). Take3 micro-volume plate and Synergy MX plate reader were both calibrated for absorbance based on manufacturer’s instructions. Estimated extinction coefficients and molecular weight were calculated using Integrated DNA Technologies’ OligoAnalyzer 3.1 (Coralville, IA; www.idtdna.com/calc/analyzer, Table 1)[18]. A new synthetic oligonucleotide miRNA concentration in ng/μL was calculated based on these UV absorption measurements and extinction coefficients (Table 2). Purity of synthetic oligonucleotide miRNA was measured using the RNA 6000 Nano kit and analyzed on a 2100 Bioanalyzer Instrument using their 2100 Expert Software (Agilent Technologies; Santa Clara, CA). UV measurements were normalized to microRNA Peak (% of Total) and number of copies (x 10^13^) was calculated using the molecular weight and Avogadro’s number.

**Table 2:** Measured values of synthetic RNA oligonucleotides.

Synthetic microRNA	UV Measured Concentration (ng/μL)	Mean Standard Error	Regression Coefficient	miRNA Peak (% of Total)	Normalized UV Concentration (ng/μL)	Copy Number (x10^13^)
cel-miR-238-3p	791.16	9.10	0.9991	98	775.34	6.49
cel-miR-39-5p	800.04	8.03	0.9999	99	792.04	6.88
hsa-miR-155-5p	1029.86	4.81	0.9997	53	N/A	N/A
hsa-miR-223-3p	772.19	4.91	0.9998	96	741.30	6.49

“Normalized UV concentration,” = UV Measured Concentration x miRNA Peak percentage

Total RNA extract was measured using RNA 6000 Nano kit and analyzed on a 2100 Bioanalyzer Instrument using their 2100 Expert Software (Agilent Technologies; Santa Clara, CA). Cell-associated total RNA concentration was calculated based on extinction coefficient and other proprietary formulations built into the Bioanalyzer 2100 and 2100 Expert Software (Agilent Technologies; Santa Clara, CA).

### cDNA synthesis

TaqMan Small RNA Assays Kits, containing both cDNA synthesis enzymes, buffers and gene-specific primers (Table 3) were purchased from Thermo Fisher Scientific (Grand Island, NY). cDNA was synthesized based on the manufacturer’s instructions with the following amendments: total reaction volume was halved by proportionally dividing all components and reverse transcriptase was added individually to reaction tubes. Primers were used at suggested concentrations for reverse transcription.

**Table 3:** General information about microRNAs.

Kit	Target	Accession Number	PCR Primer Dilution (Factor of X)	Annealing Temperature (^o^C)
Exiqon	cel-miR-238-3p	MIMAT0000293	20	61 to 62
	cel-miR-39-5p	MIMAT0020306		
	hsa-miR-155-5p	MIMAT0000646		
	hsa-miR-223-3p	MIMAT0000280		
Qiagen	cel-miR-238-3p	MIMAT0000293	40	55 to 56
	cel-miR-39-5p	MIMAT0020306		
	hsa-miR-155-5p	MIMAT0000646		
	hsa-miR-223-3p	MIMAT0000280		
Quanta Biosciences	cel-miR-238-3p	MIMAT0000293	67	53 to 54
	cel-miR-39-5p	MIMAT0020306		
	hsa-miR-155-5p	MIMAT0000646		
	hsa-miR-223-3p	MIMAT0000280		
Thermo Fisher Scientific	cel-miR-238-3p	MIMAT0000293	20	58 to 60
	cel-miR-39-5p	MIMAT0020306		
	hsa-miR-155-5p	MIMAT0000646		
	hsa-miR-223-3p	MIMAT0000280		

QScript miRNA cDNA Synthesis Kit was purchased from Quanta Biosciences (Beverly, MA) and cDNA synthesis was (Fig 1B) based on manufacturer’s instructions with the following amendments: total reaction volume was halved by proportionally dividing all components and reverse transcription temperature was decreased to 37°C.

Universal cDNA Synthesis Kit II, containing miRCURY LNA miRNA PCR, Polyadenylation and cDNA synthesis kit II, was purchased from Exiqon (Vedbaek, Denmark), miScript II Reverse Transcription Kit was purchased from Qiagen (Hilden, Germany) and cDNA was synthesized (Fig 1B). Both kits were only modified from manufacturer’s instructions by dividing reaction volume and respective components in half.

cDNA controls were made for all kits by omitting the components as described in results section. Volumes and components were proportionally decreased in all instances to reduce the amount of template sample needed.

### PCR annealing temperature validation

cDNA was amplified using AmpliTaq Gold, PCR Buffer, Magnesium Chloride Solution, dNTPs, (Thermo Fisher Scientific; Grand Island, NY) and primers for each respective kit (Table 3). Primers were purchased from all respective companies, except in the case of primers associated with Quanta Bioscience. Quanta Bioscience primers were developed based on manufacture’s guidance and purchased from Integrated DNA Technologies (Coralville, IA).

Appropriate engineering and manual controls were used to prevent contamination including: master mix made using a clean hood prior to adding any template, clean gloves, and PCR-clean reagents and consumables. Concentrations of components, cycle conditions, including initial annealing temperature ranges, were chosen based on manufacturer’s recommendations. Amplicons were analyzed using the FlashGel System (Lonza; Basel, Switzerland) per manufacturer’s instructions for small amplicons.

### Droplet digital PCR

For all experiments indicated below, a master mix was initially made containing all respective components, except template, in a clean hood within a PCR-sterile room (Fig 1C). Reaction master mixes were aliquoted in a separate room and template was added using sterile techniques.

Corresponding primer dilutions are indicated (Table 3). Primers for Thermo Fisher Scientific, Qiagen, and Exiqon are proprietary and therefore dilution factors are relative to manufacturer’s stock. Initial concentrations for Quanta Bioscience Primers were 10 μmol/L.

For Thermo Fisher Scientific reactions, Supermix for Probes containing dUTP (Bio-Rad; Hercules, CA) was used in the ddPCR master mix. cDNA from the TaqMan Small RNA Assay Kit was serially diluted in water and added as technical replicates. Droplets were generated using Bio-Rad’s manual droplet generator and droplet generating oil for probes (Bio-Rad; Hercules, CA).

For Exiqon, Qiagen, and Quanta Bioscience experiments, a ddPCR master mix containing respective primers (Table 3) and Bio-Rad EvaGreen Supermix (Bio-Rad; Hercules, CA) was made. cDNA from Exiqon cDNA Synthesis Kit II, Qiagen’s miScript Kit, or Quanta Bioscience’s qScript kit, were individually diluted serially into water and added to respective reactions as technical replicates. Droplets were generated using Bio-Rad’s manual droplet generator and EvaGreen droplet generating oil (Bio-Rad; Hercules, CA).

Droplets were hardened using cycling conditions shown in Table 4. The annealing temperatures are based on the results from annealing temperature optimization studies.

**Table 4:** Droplet digital cycling conditions.

EvaGreen Supermix				Supermix for Probes containing dUTP			
Cycling Step	Temperature (°C)	Time (min)	# of Cycles^a^	Cycling Step	Temperature (°C)	Time (min)	# of Cycles^a^
Enzyme Activation	95	5	1	Enzyme Activation	95	10	1
Denaturation	95	0.5	40	Denaturation	94	0.5	45
Annealing/ Extension	See Table 1	1		Annealing/ Extension	See Table 1	1	
Signal Stabilization	4	5	1	Enzyme Deactivation	98	10	1
	90	5	1				
Hold	4	5 to ∞	1	Hold	4	5 to ∞	1

^a^Ramp rate is 2°C/s.

All droplets were analyzed using the QX200 Droplet Digital System (Bio Rad; Hercules, CA). Data was acquired using 1-dimensional or 2-dimensional based plotting systems as recommended by the manufacturer. Thresholds were set by excluding only the true negative population [16].

### Statistical analysis

Data was exported from each relative manufacturer’s software into a spreadsheet-based analysis system where appropriate. Data was analyzed, graphed and correlated using either GraphPad Prism, R Statistical Programming using RStudio [19, 20], or Microsoft Excel. Specific graphical or statistical analysis software is indicated in the figure legends.

## Results

### Measurement of synthetic oligonucleotides

Synthetic oligonucleotides are a convenient and easy way to optimize reaction conditions while simultaneously controlling multiple variables [13]. We began by choosing two non-homologous and two homologous human miRNAs to evaluate. For our non-human miRNA we choose two *Caenorhabditis elegans* (cel) miRNA homologs, cel-miR-238 and cel-miR-39 (Table 3) [21]. For miRNAs homologous to *Homo sapiens* (hsa), we choose two miRNAs implicated in different disease etiologies that could be traced to specific cell subsets. Hsa-miR-155 and hsa-miR-223 have been shown to be differentiated in multiple types of diseases [22–24]. It has been shown that hsa-miR-155 plays a role in macrophage polarization [25] and that hsa-miR-223 has a role in neutrophil function [26].

**Fig 2:**
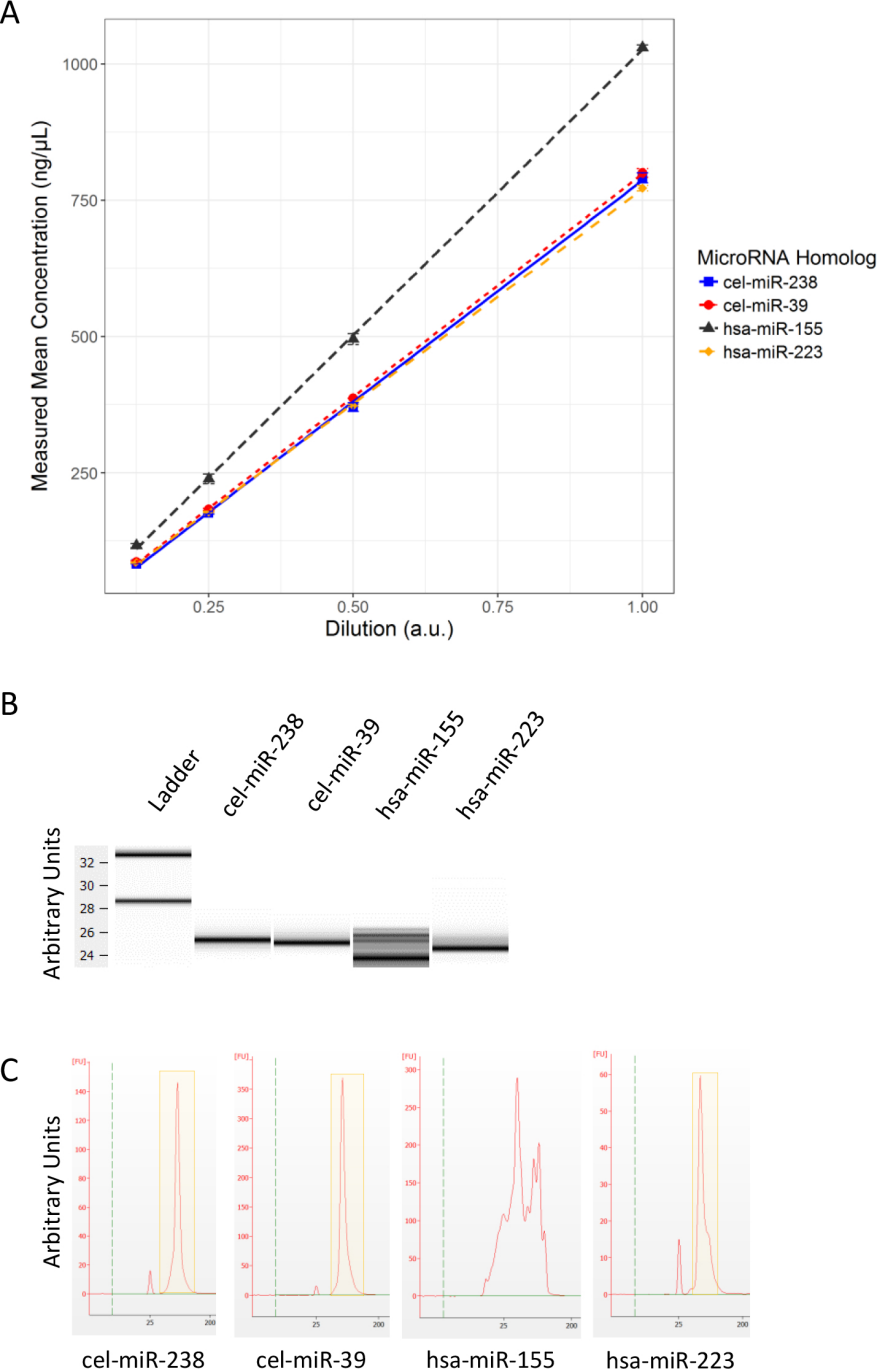
Analytical data for synthetic oligonucleotides. Manufacturer reported that synthetic oligonucleotides had a specific molecular weight and diluting them as instructed would yield a concentration of 100 μmol/L. A microvolume UV spectrophotometer was used to acquire absorbance data for four synthetic oligonucleotides and (A) graphed as mean measured concentration versus manufacturer intended concentration. Data from the chip-based automated electrophoresis system is shown as (B) electronic gel or (C) individual spectral data. Yellow box on electropherograms represents observed individual peaks corresponding to synthetic miRNA oligonucleotides. Graph (A) was developed using ggplot2 in RStudio. Imagines (B) and (C) are directly from 2100 Expert Software (Agilent Technologies; Santa Clara, CA).

**Fig 3:**
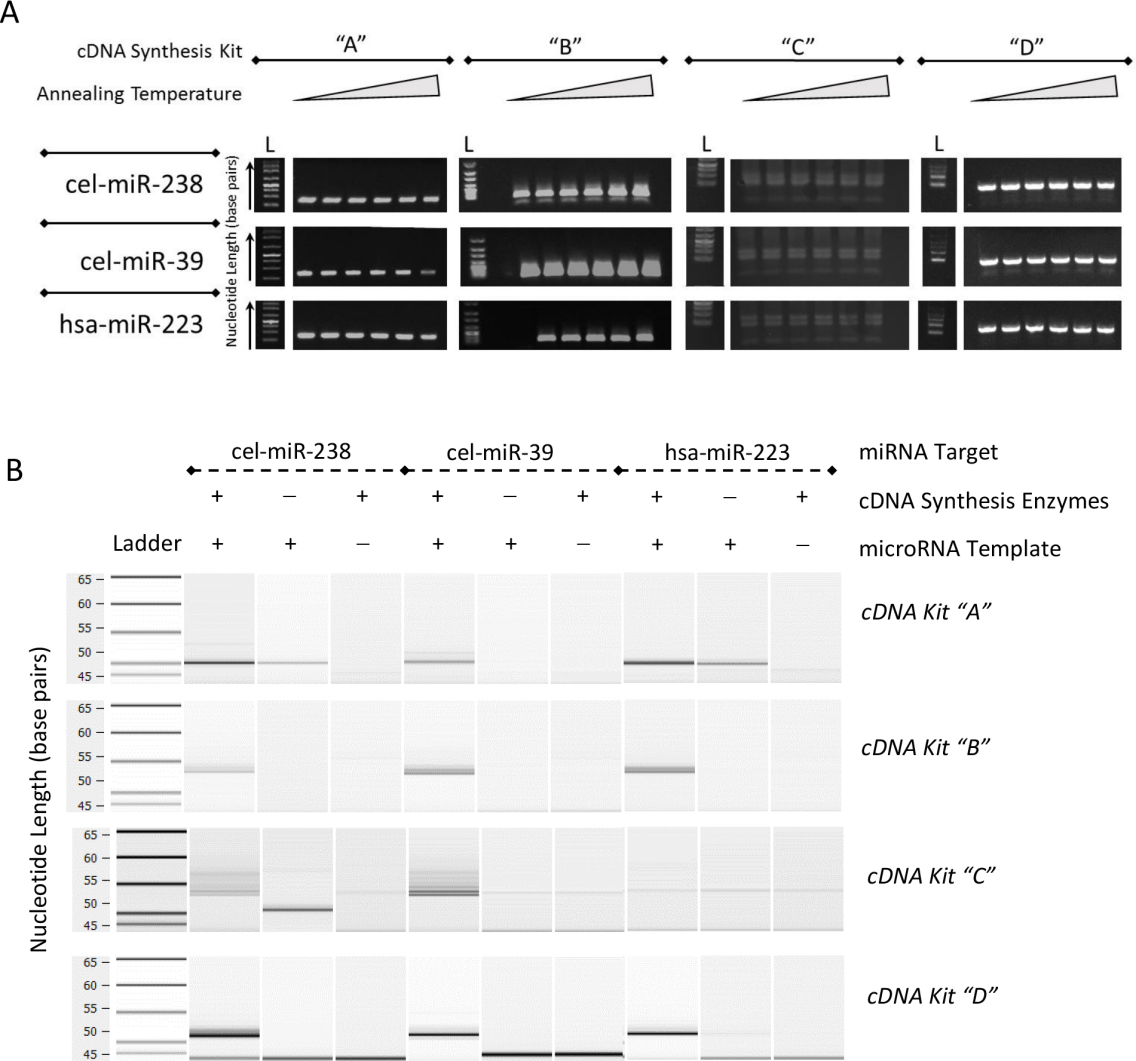
Annealing temperature evaluation using PCR. (A) Traditional gel electrophoresis pictures after polymerase chain reaction (PCR) with increasing annealing temperatures done after cDNA synthesis with cDNA Kits”A”, “B”, “C”, or “D”. (B) Chip-based automated electrophoresis system gel image of cDNA amplified using PCR. Annealing temperature ranges as indicated per kit in degree Celsius: cDNA Kit “A” = 52, 56, 58, 60, 62, 65; cDNA Kit “B” = 50, 52, 55, 58, 60, 62; cDNA Kit “C” = 50, 53, 56, 58, 60, 62; cDNA Kit “D” = 52, 56, 58, 60, 62, 65. “L” indicates a ladder lane. Synthetic oligonucleotide targets are listed on left by homologous nucleotide sequence.

**Fig 4:**
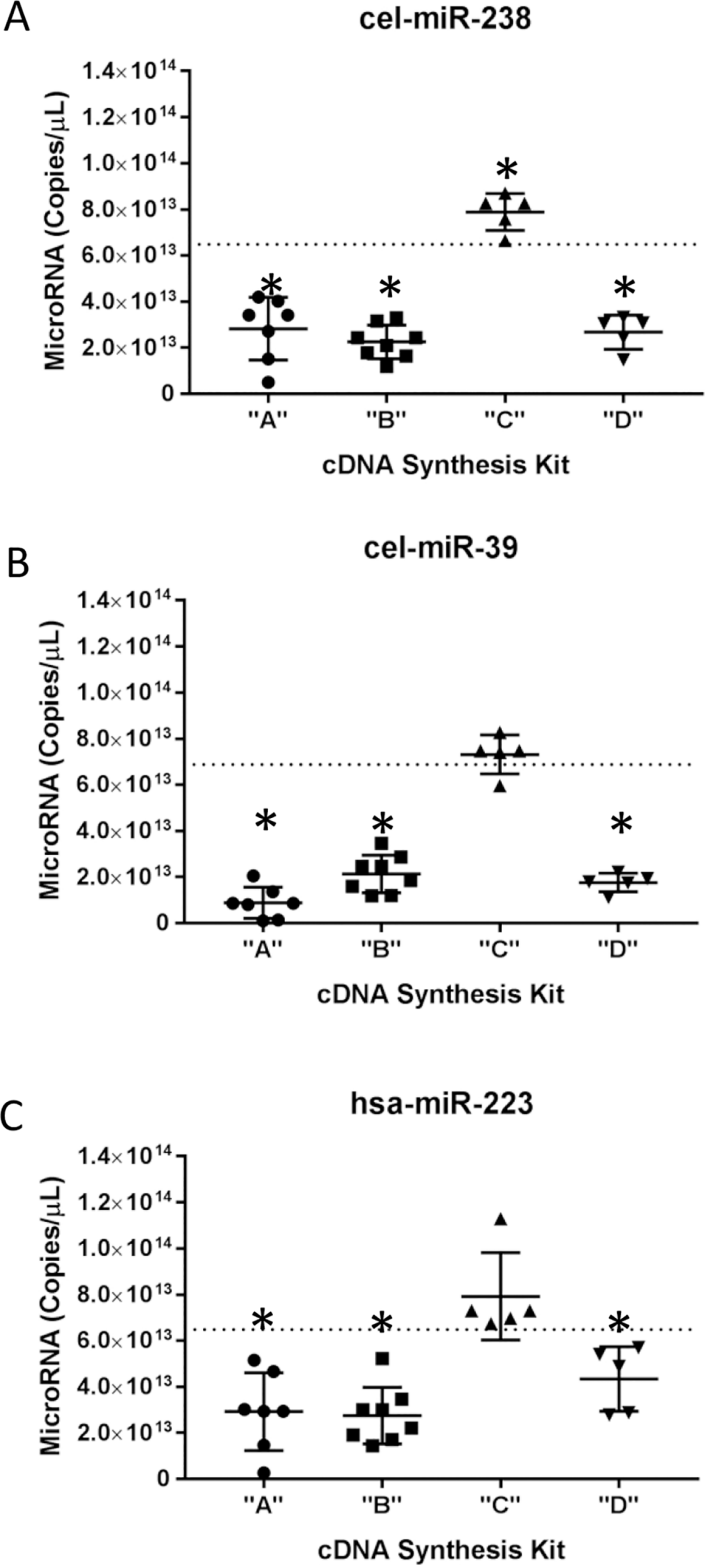
Quantification of synthetic miRNA using four different cDNA synthesis kits. (A) cel-miR-238, (B) cel-miR-39, and (C) hsa-miR-223 were measured using ddPCR following cDNA synthesis with cDNA synthesis kit “A”, “B”, “C”, or “D”. Multiple technical replicates were performed over a series of months and data was normalized for no template control and no enzyme control. Final data was graphed as miRNA copies/μL master mix. Predicted values were estimated based on UV absorbance values and chip-based automated electrophoresis system analysis. Dotted line represents predicted value. Each individual point represents a mean for a specific experimental date. The measurement uncertainty is graphed on each data set. Graphs were developed and analyzed using GraphPad Prism. (*) implies that there is 95% confidence that the measured value for an individual dataset is significantly different then the hypothetical predicted value as indicated by the dotted line.

**Fig 5:**
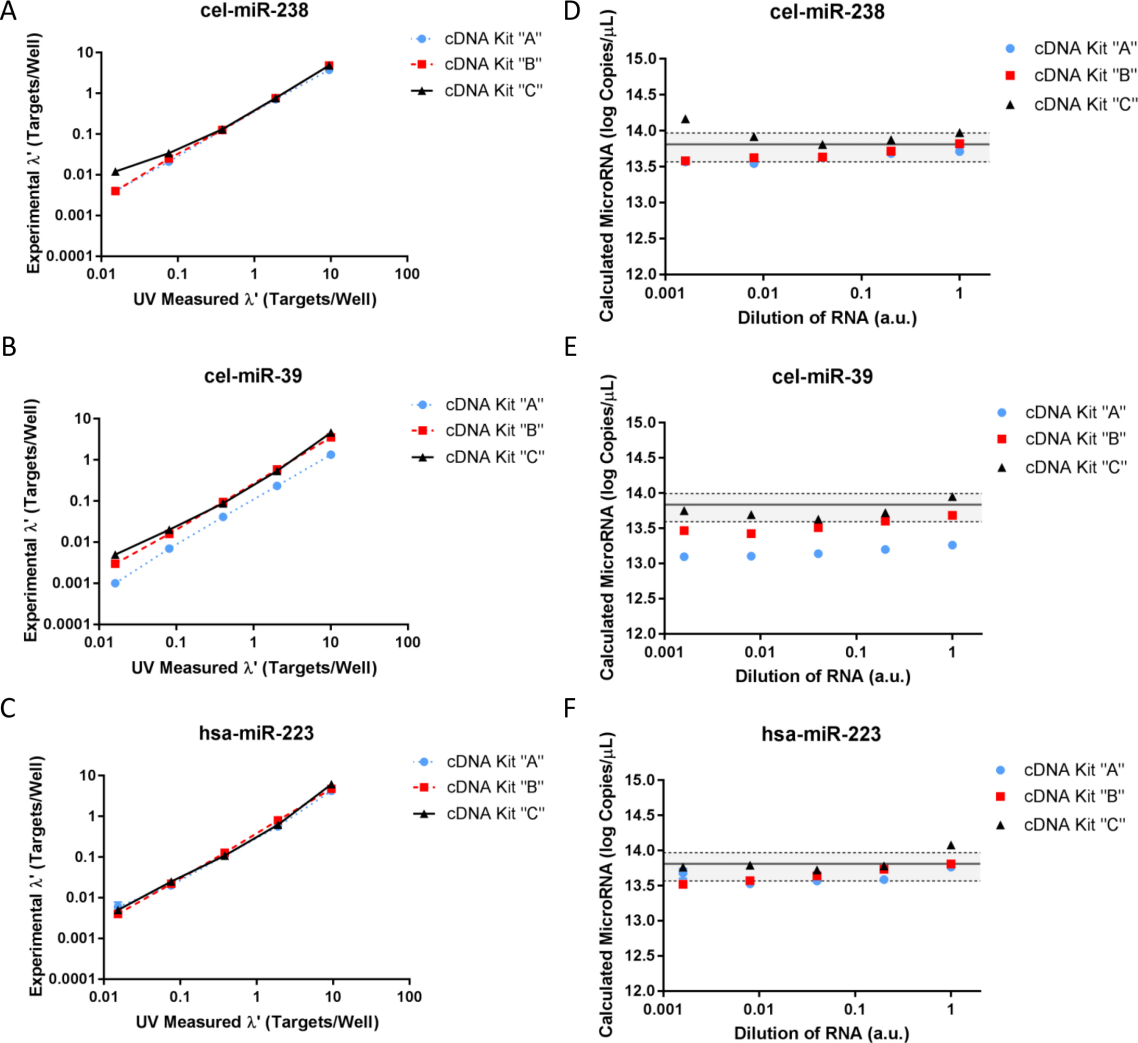
miRNA titration curves. All synthetic oligonucleotides were combined into a target master mix and subsequently diluted prior to cDNA synthesis. cDNA master mix was minimally diluted prior to ddPCR quantification. Values were normalized by subtracting average NTC and NEC from total positive droplets per kit. All three cDNA synthesis kits, “A”, “B”, and “C” graphs were organized by three targets: (A, D) cel-miR-238, (B, E) cel-miR-39, or (C, F) hsa-miR-223. cDNA synthesis kits were used to synthesize cDNA and data were plotted as (A to C) UV measured as determined by dilution factor times UV measured versus normalized experimental targets per well. (D to F) Normalized targets per well were extrapolated to miRNA copies/μL by multiplying by droplet volume, cDNA dilution factor, miRNA dilution factor, dilution into master mix, and molecular weight. The predicted copies of miRNA (Table 2) is shown as a black line. Gray shading indicates the 95% coverage interval for predicted miRNA copy number. miRNA dilutions are reported in arbitrary units and correspond to 1×, 0.2×, 0.04×, 0.008×, and 0.0016×. The standard uncertainty is shown for each point, a result of three technical replicates for one experiment. Graphs are shown as log-log scale for visualization; they were developed using GraphPad Prism. Statics were estimated using RStudio.

**Fig 6:**
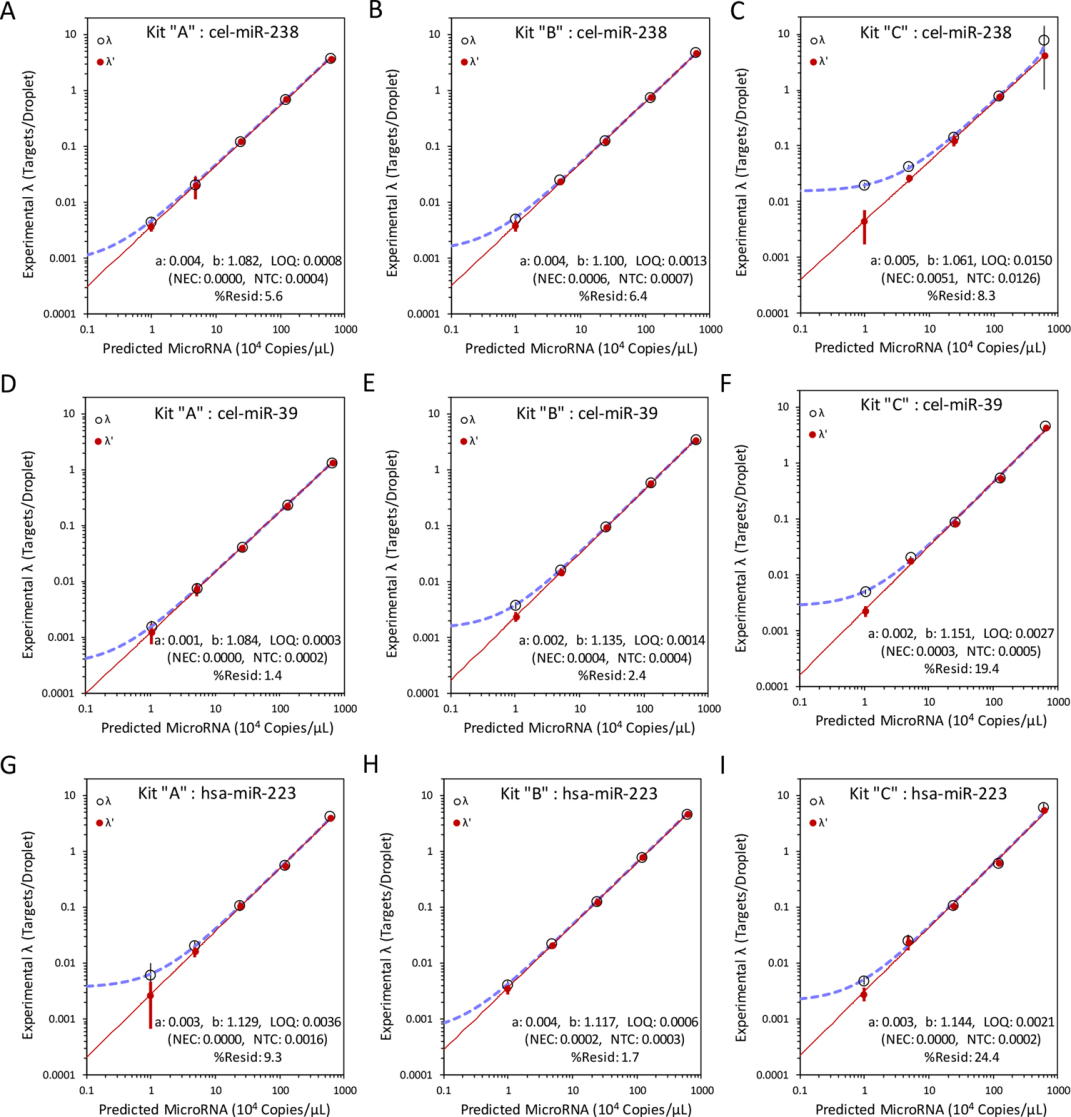
Model for miRNA quantification using synthetic oligonucleotides. miRNA titrations were performed with (A, D, G) cDNA Kit “A”, (B, E, H) cDNA Kit “B”, and (C, F, I) cDNA Kit “C”. The cDNA synthesis kits and targets were measured with droplet digital PCR system. Average no template control (NTC) and no enzyme control (NEC) were similarly measured for each experiment. Fraction positive targets per droplet were calculated either without subtracting average NEC and NTC positives (λ; open circles, purple line) or with subtracting NEC and NTC positive (λ’; closed red circles, red line) droplets. For each kit and each target (A to C) cel-miR-238, (D to F) cel-miR-39, and (G to I) hsa-miR-223, both λ (dashed purple line) or λ’ (solid red line) were graphed on a single *x*-axis versus estimated 10^4^ miRNA copies/μL master mix. A simple power curve, *y* = a*x*^b^, was calculated for normalized data, λ’. Limit of quantification (LOQ) was predicted based on the lowest possible value that satisfied the power curve. Individual data points are shown with corresponding standard uncertainties. Microsoft Excel was used to calculate and graph data.

In later experiments, the non-homologous miRNAs would be used as spike-in controls and the miRNAs homologous to *Homo sapiens* would be measured endogenously in samples of interest. It is important to computationally check non-homologous spike-in sequence homology to human miRNA and identify any places where the spike-in controls might hybridize to human mRNA, as well as run a wet-lab validation study with a non-precious sample to identify limit of detection (LOD). We used miR-Base [17] to identify the sequence of each miRNA (Table 1) and confirm that they did not have sequence homology to other miRNA in humans or other species. Additionally, a validation study entails quantifying off-target effects in total RNA purified from a non-precious sample that does not have any exogenous synthetic miRNA spiked into it. Although these off-target effects can be hypothesized using bioinformatics it is important to determine an experimental value upfront to assess if maybe a different spike-in target could be used (S2 Fig). Lastly, it was important for economic and practical reasons to use miRNAs that were compatible with readily available and cost-effective primers and probes. For initial experiments (Figs 2–6), synthetic oligonucleotides were created, purchased, tested, and utilized for all targets of interest.

Upon receiving the synthetic oligonucleotides, we measured the absorbance using a microvolume compatible plate reader. UV spectrophotometer measurements of nucleic material has recognized inherent issues. However, we often use it because it serves as a good benchmark for concentration measurement [27]. The purpose for its use in this study is as a relative method for quantitative analysis of miRNA.

Absorbance values were measured at four different dilutions for each oligonucleotide. Values were converted to concentrations of nanograms per microliter for reference and plotted against their dilution values (Fig 2A). Using R statistical programming [19], we calculated the mean concentration, uncertainty, and regression coefficient of each synthetic oligonucleotide dilution. All values had small uncertainties and the only deviation from expected concentration, based on manufacturer’s estimated concentration, was hsa-miR-155 (Table 2). To confirm that our synthetic oligonucleotides were free of partial products or solubilized impurities we evaluated each oligomer on a chip-based automated electrophoresis system.

Both gel (Fig 2B) and spectral data (Fig 2C) demonstrated clean bands and sharp peaks, respectively, for all oligonucleotides apart from hsa-miR-155. Hsa-miR-155 showed multiple bands and a wide distribution of apparent molecular weights. The chip-based automated electrophoresis system software uses their ladder as a standard for concentration determination by comparing areas under the peaks of the ladder to those of the sample. Furthermore, the RNA integrity number (RIN) is calculated using ribosome RNA, which is not present in our synthetic miRNA oligonucleotides. miRNA peak as percentage of total peak was calculated by drawing a nominal region around the single peak within the electropherogram (Fig 2C, yellow box). We attempted to normalize concentrations obtained using absorbance measurements for all oligonucleotides by multiplying the concentration determined by UV absorbance and specific miRNA percentage of total area of spectral data representing pure miRNA (Table 2). Since there is no single hsa-miR-155 peak, we could not definitively identify the percentage of pure full-length miRNA product in the stock.

We believe that the impurities associated with our synthetic hsa-miR-155 oligonucleotide may not be characteristic of endogenously expressed hsa-miR-155. However, the heterogeneity of the hsa-miR-155 synthetic oligonucleotide makes accurate quantification difficult, so we excluded exogenous measurements for hsa-miR-155 in all remaining technical exogenous validation experiments (Figs 3–6). Instead, we focused on evaluating only cel-miR-238, cel-miR-39, and hsa-miR-223 as assay controls. We use these normalized UV concentration values (Table 2) for cel-miR-238, cel-miR-39, and hsa-miR-223 throughout the paper.

### Optimizing polymerase chain reaction conditions

Primer designing is a skill that uses fine-tuned principles to predict the best oligonucleotide to specifically hybridize to the target of interest [28]. Unfortunately, most reagents used in two-step ddPCR for miRNA quantification are proprietary, which complicates conventional primer design techniques. For example, adaptor sequences, which are the oligonucleotide extensions added to miRNA cDNA, are used so that targets are long enough for primer hybridization. Depending on the miRNA cDNA Synthesis kit, a general polyA tail, hairpin loop, a universal tag sequence, or a combination of technologies are added to the end of the cDNA either before or during reverse transcription. These technologies and their proprietary nature make identifying the true length of the intended products nearly impossible and create ambiguity when trying to optimize hybridization conditions. An additional complication of using conventional primer design is that the native mature miRNA targets are only 21 to 24 nucleotides in length [17] so there is a limited choice of hybridization sites that optimize specificity. These two substantial obstacles leave an investigator with limited ability to apply traditional primer and probe optimization strategies.

We began by performing a standard annealing temperature experiment to determine if annealing temperature had any effect on non-specific products and amplification efficacy (Fig 3A) [29]. In addition to full length targets, non-specific products could also be observed at other molecular lengths at all annealing temperatures for all kits (Table 5). The source of these varying molecular weight products was investigated using different negative controls. We recognized early in our experiments that the traditional no template control, which simply omits RNA template prior to cDNA synthesis would not be sufficient [15]. We therefore postulated that there were a variety of different negative controls available for these kits, including (1) omitting RNA prior to cDNA synthesis, (2) omitting enzymes involved in cDNA synthesis, (3) omitting cDNA in ddPCR reaction, (4) omitting Supermix for the ddPCR reaction, and (5) omitting primers or probes at any level. We found that omitting Supermix or primers very rarely gave any positive droplets and attribute the few positive droplet occurrences to technician contamination. When we omitted cDNA in the annealing temperature PCR experiment, the abundance of non-specific byproducts tended to correlate with the amount and size of primers (Table 5). For example, cDNA Kit “D”, which alone uses primers in both the cDNA synthesis step and ddPCR step (Fig 1B) has double the number of non-specific bands for target cel-miR-238 compared to any other kit. Consequently, we decided that these three controls are valuable to run occasionally, but are not essential for every reaction given limited sample and reagent availability. Therefore, it is our recommendation to use the following controls: (1) omitting RNA prior to cDNA synthesis, which we can call a true no template control (NTC) and (2) omitting enzymes involved in cDNA Synthesis, which can be called no enzyme control (NEC).

**Table 5:** Non-specific bands in control samples at different annealing temperatures.

		Annealing Temperatures (°C)					
**cDNA Kit (A)**	**Target**	**52**	**56**	**58**	**60**	**62**	**65**
(-) Reverse Transcriptase (NEC)	cel-miR-238					+	
	cel-miR-39						
	hsa-miR-223						
(-) RNA Template (NTC)	cel-miR-238	+++	+++	+++	+++	++	
	cel-miR-39	+					
	hsa-miR-223						
(-) cDNA Template	cel-miR-238						
	cel-miR-39						
	hsa-miR-223						
**cDNA Kit (B)**	**Target**	**50**	**52**	**55**	**58**	**60**	**62**
(-) Reverse Transcriptase (NEC)	cel-miR-238	++	++	++	++	++	++
	cel-miR-39	+	+	+	+	+	+
	hsa-miR-223			+			
(-) RNA Template (NTC)	cel-miR-238	++	++	++	++	++	++
	cel-miR-39	++	++	++	++	++	++
	hsa-miR-223	++	++	++	++	++	++
(-) cDNA Template	cel-miR-238	+	+	+	+	+	+
	cel-miR-39	+	+	+	+	+	+
	hsa-miR-223	+	+	+	+	+	+
**cDNA Kit (C)**	**Target**	**50**	**53**	**56**	**58**	**60**	**62**
(-) Reverse Transcriptase (NEC)	cel-miR-238	+	+	+	+	+	+
	cel-miR-39	+	+	+	+	+	+
	hsa-miR-223	+	++	+	+	+	+
(-) Poly(A)Polymerase (NEC)	cel-miR-238	+++	+++	+++	+++	+++	+++
	cel-miR-39	+++	+++	+++	+++	+++	+++
	hsa-miR-223	+++	+++	+++	+++	+++	+++
(-) RNA Template (NTC)	cel-miR-238	+	+	+	+	+	+
	cel-miR-39	++	++	++	++	++	++
	hsa-miR-223	++	++	++	++	++	++
**cDNA Kit (D)**	**Target**	**52**	**56**	**58**	**60**	**62**	**65**
(-) Reverse Transcriptase (NEC)	cel-miR-238	++	++	++	++	++	++
	cel-miR-39	+	+	+	+	+	+
	hsa-miR-223						
(-) RNA Template (NTC)	cel-miR-238	++	++	+++	+++	+++	++
	cel-miR-39	++	++	++	++	++	++
	hsa-miR-223					++	++
(-) cDNA Template	cel-miR-238	++	++	++	++	++	++
	cel-miR-39	+	+	+	+	+	+
	hsa-miR-223						

+, single band or smear present.

++, double band present.

+++ triple band present.

In NTC and NEC reactions, non-specific products were observed in all cDNA Synthesis kits depending on the temperature and target. Table 5 lists instances where non-specific bands were observed for NTC and NEC controls. Each positive sign indicates one various size non-specific band. Some bands were hypothesized to be primers, but others corresponded to full length targets. For example, a band of approximate target cDNA size was seen in each of the following reactions: with cDNA Kit “A”, at cel-miR-39, in the NTC; with cDNA Kit “B” at cel-miR-238, in the NEC; with cDNA Kit “C” at hsa-miR-223, in the Poly(A)Polymerase NEC; and with cDNA Kit “D”, at cel-miR-238, in the NTC. Using these conventional PCR annealing temperature analyses we chose an optimal annealing temperature that would minimize as many non-specific products as possible (Table 3).

We also explored the effect of temperature on the reverse transcription step for the cDNA Kit “C”. In this kit, reactants are added in two different steps: first poly(A) polymerase and reaction buffers are added for tail extension to the template and then reverse transcriptase is added for cDNA synthesis (Fig 1B). All other kits contain enzymatic mixtures with all components and therefore restrict ability to test non-specific product formation resulting from a singular enzyme and a single cycle reaction. The manufacturer of cDNA Kit “C” recommends performing the polyA extension at 37°C and reverse transcription at 42°C. However, given that all other kits used similar conditions for both adaptor extension and reverse transcription, we tested different reverse transcriptase temperatures to see if we could standardize temperature for both enzymatic reactions. We found no specific differences when using either 37°C or 42°C for reverse transcription when examined using gel electrophoresis so we choose to perform both enzymatic reactions at 37°C.

In a further effort to check specificity and purity, we also evaluated PCR-amplified products using a chip-based automated electrophoresis system (Fig 3B). We used the optimized annealing temperatures for all reactions. Non-specific products were seen under most conditions, often with varying sizes. All kits showed at least one non-specific product in NTC or NEC. These non-specific products have the potential to skew the precision of the quantitative measurements. Therefore, we decided to do repeated tests evaluating technical variation in experimental miRNA copy number data when quantifying synthetic oligonucleotides using ddPCR.

### Relative concentration and copy numbers for kits and targets

We performed multiple experiments using a mixture of miRNA synthetic oligonucleotides, generally at equal ratios to each other. In each case, the miRNA synthetic oligonucleotides were diluted approximately 10^5^ to 10^9^ fold and synthesized into single stranded cDNA (Fig 1B) as shown in our schematic in S1 Fig. Following reverse transcription, the cDNA was diluted to a nominal concentration and copy number measured using a ddPCR system (Fig 1C). For these experiments, cDNA was stored at 4°C or -20°C based on manufacturer’s instructions, cDNA mixtures were evaluated using ddPCR anytime between zero and five days following cDNA synthesis. However we suggest storing cDNA at 4°C. Storage at 4°C, especially in instances where multiple freeze-thaws might occur, has been shown to reduce DNA accumulation on the sides of storage container and improve recovery [27].

Fig 4 shows experimentally-derived miRNA copy number for each cDNA synthesis Kit. The dashed line represents predicted miRNA copy number as derived in previous experiments (Fig 2 and Table 2). All samples were normalized to controls by subtracting the average fraction positive NTC and NEC from sample fraction positive and then by applying Poisson’s distribution directly to the fraction negative as calculated by one minus fraction positive (Fig 1D). This value is the normalized copies per droplet (λ’).

These preliminary data demonstrate that the mean measured miRNA copy number in cDNA Kits “A”, “B”, and “D” is significantly different (p < 0.05) from the predicted miRNA copy number for all three targets (Fig 4). Conversely, although certain measurements for cDNA Kit “C” were higher than the predicted copy number, only the mean measured cel-miR-238 copy number was significantly different from predicted copy number (Fig 4A). Each test was done by using a one-sample t test comparing each actual mean to theoretical mean after having passed the Shapiro-Wilk normality test [20].

We compared both the repeatability (the spread of technical replications over different days) and the average copy number detected in all trials. cDNA Kit “C” has the lowest coefficient of variation for all measurements of target concentration with 10.3%, 11.5%, and 23.9% for cel-miR-238 (Fig 4A), cel-miR-39 (Fig 4B), and hsa-miR-223 (Fig 4C), respectively. However, cDNA Kit “B” has the lowest standard uncertainty for both cel-miR-238 (Fig 4A) and hsa-miR-223 (Fig 4C), at 2.58 × 10^12^ miRNA copies per microliter (copies/μL) and 4.34 × 10^12^ copies/μL, respectively. Whereas the uncertainty for cDNA Kit “D” is 1.80 × 10^12^ copies/μL for cel-miR-39 (Fig 4B). Therefore, we conclude that all kits show some marginal target-specific repeatability discrepancies.

Our results are in accordance with other metrology institutes that reported similar findings depending on the target and kit [11]. We further extrapolated these data to create an accurate measurement model that can be used to quantitatively measure any miRNA copy number, agnostic of cDNA synthesis kit preference. We began by controlling our experiments for variables to allow for a fair comparison. For example, because cDNA Kit “D” uses gene specific primers in the cDNA synthesis kit, is only compatible with a different ddPCR supermix from the other three kits, did not produce significantly better results, and is cost prohibitive to use for high throughput experiments, we concentrated all future experiments and results on cDNA Kits “A” to “C”. Next, to quantitatively measure miRNA copy number, a titration curve needs to be run using validated (Figs 2 and 3) synthetic oligonucleotides homologous to the control miRNAs (Fig 1C). These data then can be applied to any clinical sample to accurately measure copy number for any miRNA of interest (Fig 1D).

### Titration of miRNA for ddPCR quantification

Reverse transcription is not a concerted reaction [6, 9]. Therefore, to quantify the limit of quantification, estimate non-specific positive droplets, and create a formula to convert experimentally measured miRNA to predicted values, we performed a miRNA titration assay. This assay assumes there will be a tangible loss within the steps to measure miRNA (Fig 1, panels A to C), but the goal is to quantify units of miRNA with the least uncertainty.

We choose to measure the miRNA as copies/μL. The droplet digital PCR computes droplets with florescence as positive and droplet without florescence as negative. A fraction negative is calculated and Poisson’s correction is applied to compute targets per ddPCR droplet volume. We measured the microliter volume and its approximate 95% coverage interval of Evagreen Supermix droplets as (0.783 ± 0.018) nL [30]. Dividing the number of targets per droplet by the microliters per droplet and adjusting for dilution factor yields copies miRNA per microliter (Fig 1D).

Experimental versus expected copy number are linearly related for all targets (Fig 5A–5C). Experimental targets per well versus UV measured targets per well are strongly correlated (R^2^ > 0.99) when represented by the simple power curve *y* = a*x*^b^, where “a” is the scale constant, “b” is the scaling exponent, “*x*” is a UV measurement, and “*y*” is the ddPCR result. We evaluated each calculated miRNA copies/μL for all dilutions in a series. The predicted miRNA copy number is shown with a 95% coverage interval. Cel-miR-39 has multiple data points that fall outside of this confidence interval, partially for cDNA kit “A” and “B” (Fig 5D–5F). Nevertheless, the targets appear to segregate independently with random dispersion patterns [31], which satisfies key assumptions necessary for using Poisson’s distribution to model ddPCR and further extrapolate data to report copy number. Therefore, we can use this titration data to propose a model that allows us to calculate the limit of quantification (LOQ), correlate loss, and accurately calculate miRNA copy number.

Each target, for every kit, has their own specific NTC and NEC values. We plot the raw values (λ) and the normalized values (λ’) on the same graph (Fig 6). Normalized values were calculated by subtracting NTC and NEC positives before converting data to targets per droplet (Fig 1D). Hence, by optimizing for lowest uncertainty and percent residual difference between predicted absolute and normalized copies per droplet we derive a LOQ for each target and each kit (Table 6). Additionally, we use the LOQ value and the corresponding scaling factor “a” and exponent “b” to derive the predicted limiting miRNA copy number per microliter master mix that might be detected reliably.

**Table 6:** Synthetic microRNA oligonucleotide titration for each kit and target.

Kit	Target	NEC	NTC	a	b	Limit of Quantification (targets/droplet)	Limit of Quantification (10^4^ microRNA copies/μL)
(A)	cel-miR-238	0.0000	0.0001	0.004	1.082	0.001	0.24
(A)	cel-miR-39	0.0000	0.0002	0.001	1.084	0.0008	0.30
(A)	hsa-miR-223	0.0000	0.0020	0.003	1.129	0.004	1.28
(B)	cel-miR-238	0.0002	0.0003	0.004	1.010	0.001	0.36
(B)	cel-miR-39	0.0004	0.0001	0.002	1.135	0.001	0.65
(B)	hsa-miR-223	0.0001	0.0002	0.004	1.117	0.001	0.19
(C)	cel-miR-238	0.0011	0.0006	0.005	1.061	0.015	3.08
(C)	cel-miR-39	0.0003	0.0002	0.002	1.151	0.003	1.17
(C)	hsa-miR-223	0.0001	0.0001	0.003	1.144	0.002	0.71

### Application of principles for clinical quantification of miRNA

**Fig 7:**
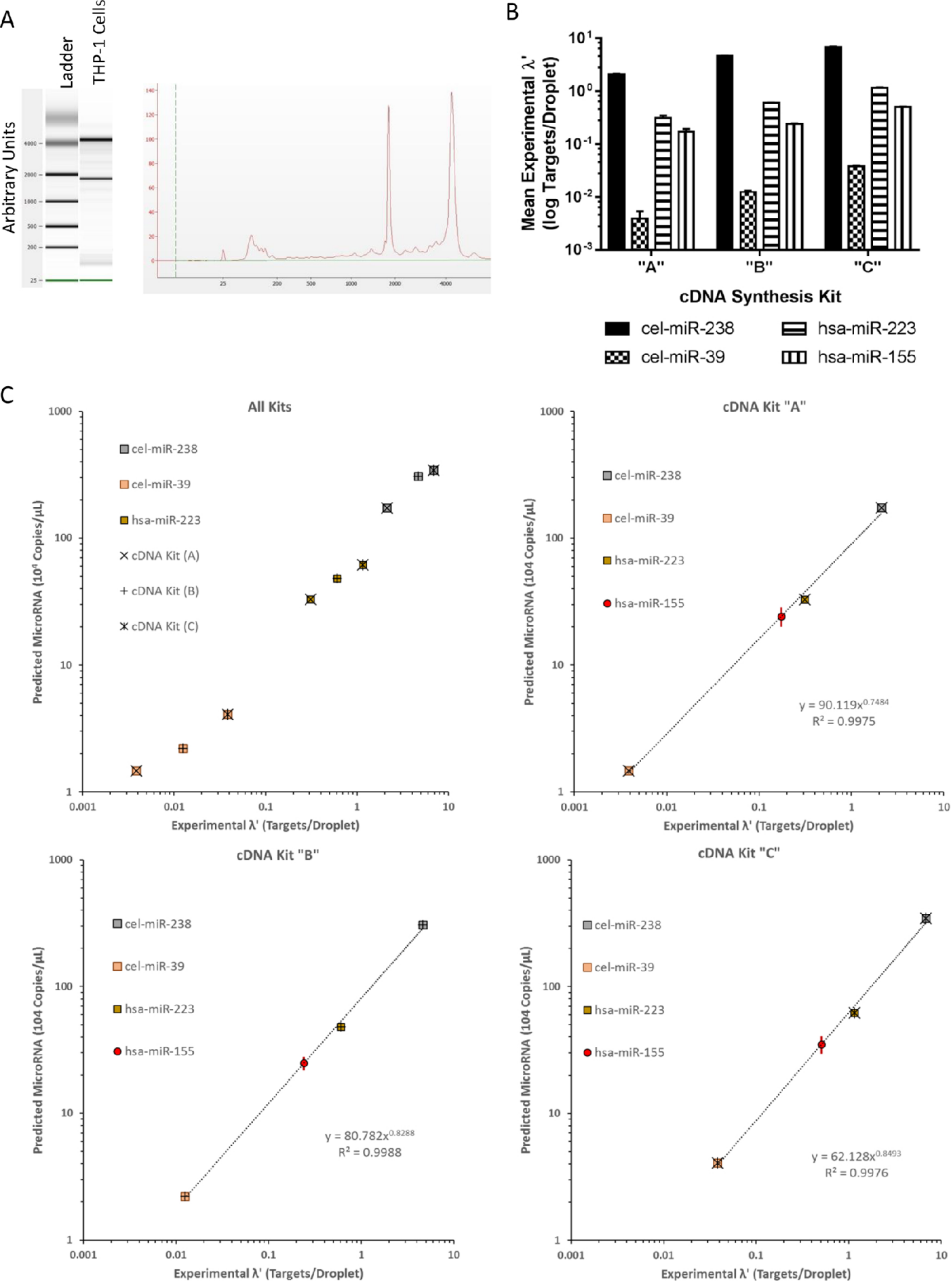
Quantitative miRNA measurements in THP-1 cells. Total RNA was extracted from macrophage derived THP-1 cells and was (A) analyzed for integrity, purity, and concentration using the chip-based automated electrophoresis system. (B) Total RNA was reverse transcribed by cDNA synthesis kit “A”, “B”, or “C” and specific miRNA targets were measured using ddPCR. (C) Each kit, “A” (×), “B” (+), and”C” (ӿ), was used to make cDNA and total number of targets were counted via ddPCR. Average NTC and NEC were similarly measured for each experiment. For all targets, cel-miR-238 (gray), cel-miR-39 (orange), hsa-miR-223 (brown), and hsa-miR-155 (red), fraction positive targets per droplet were calculated by subtracting NEC and NTC positive and applying Poisson distribution. Predicted 10^4^ miRNA copies/μL for cel-miR-238 (gray square), cel-miR-39 (orange square), and hsa-miR-223 (brown square) was calculated using pre-existing power curve values (Table 6). For each respective kit, a new power curve was developed using experimental versus predicted cel-mir-238, cel-miR-39, and hsa-miR-223 concentration (Table 7). This new power curve was applied to respective, endogenously measured, experimental hsa-miR-155 λ’ to generate predicted hsa-miR-155 (red circle) copies/μL. The standard uncertainty is shown on the graph for hsa-miR-155 for each respective point (dark red line). For visualization purposes, data is either graphed on one chart or separated out based on cDNA synthesis kit. New power models are calculated only for individual cDNA synthesis kits and both equation and correlation coefficient are interlayered. Graphs are single representations of repeated biological trials. Microsoft Excel was used to calculate and graph data.

To test the quantitative miRNA measurement capabilities for clinical purposes, we took the monocytic leukemia cell line THP-1 and differentiated them into M1-like macrophages using phorbol-12-myristate-13-acetate and ultra-pure Lipopolysaccharide from *E*. *coli*. Cells were flash frozen in liquid nitrogen and stored at -80°C prior to total RNA isolation. We added two exogenous spike-in controls, cel-miR-238 and cel-miR-39. One spike-in control was added at the beginning of total RNA isolation at a maximum concentration and one was added at the end of the total RNA isolation at a minimum concentration (Fig 1A). Specifically, the synthetic cel-miR-238 oligonucleotide was spiked-in at approximately 50 nmol/L concentration and added after cell lysis and major debris clearing. The synthetic cel-miR-39 was spiked-in at approximately 50 pmol/L concentration prior to freezing total isolated RNA in individual aliquots. All spike-in concentrations were substantially above the LOD and LOQ, which minimizes uncertainty (S2 Fig). Data using the chip-based automated electrophoresis system showed a wide array of differentially-sized RNA components within our total RNA isolate (Fig 7A), with a RIN of 8.60 and total concentration of 522 ng/μL.

Using our normalization method and plotting for mean targets per droplet for each kit and target, cel-miR-238 consistently has the highest droplet counts and cel-miR-39 consistently has the lowest (Fig 7B). Endogenously expressed hsa-miR-238 and hsa-miR-155 both have counts in between these spike-in values.

Spiking-in synthetic miRNA oligonucleotides at highest and lowest ranges and testing an endogenous miRNA within that range is optimal for statistical modeling purposes. Furthermore, a minimum of three measurements are necessary to create a curve; we choose to use two spike-in controls and one endogenous marker [32]. We used the power function as depicted in Fig 1D and developed in Fig 6 to calculate predicted miRNA copy number of cel-miR-238, cel-miR-39, and hsa-miR-223, given the measured mean targets per droplet from our macrophage-derived THP-1 monocytes. We graphed the measured experimental targets per droplet versus the predicted miRNA copy number for cel-miR-238, cel-miR-39, and hsa-miR-223 (Fig 7C) and calculated a new specific power curve (Table 7; Fig 1E). This new power curve can now be applied to all other targets of interest, such as endogenously expressed hsa-miR-155.

**Table 7:** Sample specific linear power model for individual kit.

Kit	R^2^	a	b	Predicted hsa-miR-155 Copy Number (10^4^)
(A)	0.9975	90.119	0.7484	24.27 ± 4.15
(B)	0.9988	80.782	0.8288	24.85 ± 3.02
(C)	0.9976	62.128	0.8493	35.00 ± 5.42

Using the optimized annealing temperature, we measured endogenously expressed hsa-miR-155 in the THP-1 cells. Previously, we discovered that our synthetic hsa-miR-155 was impure, containing non-specific contaminates that lead to ambiguity when measuring concentration using a UV spectrophotometer (Fig 2 and Table 2). Therefore, we did not have a proper synthetic miRNA oligonucleotide to titrate hsa-miR-155. However, applying our new specific power curve for each kit (Fig 7C) we normalize experimental hsa-miR-155 concentrations to obtain an accurate estimate of miRNA copy number. The experimentally derived targets per droplet (λ’) versus miRNA copies/μL master mix are plotted on each respected graph for visualization purposes. Specific values and their relative uncertainties are shown in Table 7. The resultant is an accurate hsa-miR-155 copy number that can be used to evaluate a patient’s normal physiological state and used to prescribe or modify a drug treatment plan.

Notably, the three kits used to analyze endogenous hsa-miR-155 have different values (Table 7). Scientists at National Institute of Standards and Technology (NIST) have developed the “NIST Consensus Builder” (NICOB) [33] for summarizing replicate measurements from different laboratories, measurement methods, or experimental methods. The analysis methods included in NICOB use the uncertainty associated with these different results. We used the data from Table 7 to estimate the predicted hsa-miR-155 copy numbers. We determined that while the consensus estimate for hsa-miR-155 is 2.8 × 10^5^ copies/μL, the associated standard uncertainty is 6.5 × 10^4^ copies/μL, with a 95% coverage interval of (1.8 to 4.3) × 10^5^ copies/μL. Given this large uncertainty, more measurements are needed to determine a useful consensus value for the has-miR-155 copies/μL of master mix.

We can use these same principles to analyze cell-associated miRNA from patient peripheral blood mononuclear cells (PBMCs). We separated out CD14+ monocytes and CD66b+CD16+ neutrophils from whole blood using negative selection magnetic beads (S1 Methods). Purity of CD14+ monocyte and CD66bCD16+ neutrophil populations were confirmed using flow cytometry and hsa-miR-155 was quantified in each cell subset group. Total RNA was purified from a population of each cell subset and reverse transcribed into cDNA using cDNA Synthesis Kit “B.” Four targets, cel-miR-238, cel-miR-39, hsa-miR-155, and hsa-miR-223 were measured using ddPCR. Copy number of cel-miR-238, cel-miR-39, and hsa-miR-223 were calculated using our power curve (Fig 1D) and the experimentally derived targets per droplet (λ’) versus miRNA copies/μL master mix are plotted on each respected graph for visualization purposes. A sample-specific power curve was generated and the hsa-miR-155 experimentally derived targets per droplet (λ’) copy number was applied to calculate miRNA copies (S3 Fig).

We can normalize our predicted hsa-miR-155 copy number to the total number of cells (cell counts per extraction can be found S1 Methods), keep as per microliter, or extrapolate to mass concentration using the Avogadro constant (≈6.022 × 10^23^ copies/mol). Alternatively, within the clinic, one could convert the measured miRNA copy numbers per unit of blood. Using this method (Fig 1), an accurate miRNA copy number can be determined and extrapolated to a unit of measure that is most aptly suited for diagnosticians. Similarly, any other miRNAs can be accurately quantified using this new specific power curve and be applied in the clinical for prognostic, diagnostic, or therapeutic purposes.

## Discussion

The use of miRNA for disease detection, prognosis, outcome, and therapy is a growing market predicted to get much larger in the upcoming years [1]. However, use of miRNA in precision medicine poses quite a few challenges. Some of these challenges include: (1) disease-associated miRNA regulation has not been clearly demonstrated, meaning studies often require genome-wide screens prior to specific biomarker identification, (2) conventional measurement techniques require normalization to endogenous controls characterized by stable targets found in all tissue types at equal levels, (3) probe and primer optimization is inadequate for targets of 22 to 24 nucleotides, which is the size of mature miRNA, and (4) measurement techniques are costly and low throughput, meaning rapid clinical testing still requires significant buy-in from payers, doctors, and patients [34]. Some of these issues can be mitigated by better and more reliable measurement techniques.

Here we show that there is a cost-, time- and reagent-efficient way to quantitatively measure cell-associated miRNA with ddPCR using spike-in controls. Additionally, a proof of concept was demonstrated to measure additional endogenous miRNA resulting in increased confidence in the measurements of miRNA within a single sample. Most notably, these measurement recommendations will for the first time allow for finite miRNA copy number or concentration read-outs for a sample, independent of a disease-free or acute time point or condition.

We began by building on previous measurement concepts and studies to clarify and improve miRNA measurement techniques in individual cells. Based on manufacturer feedback, primers and probes are optimally designed by applying the Digital Minimum Information for Publication of Quantitative Real-Time PCR Experiments (MIQE) Guidelines [9], however technologies used for miRNA quantification, including primer and probes necessary for digital measurements and cDNA synthesis reagents, are proprietary and therefore severe obstacles exist for using conventional design methods. Instead, when first measuring miRNA, one must account for variation associated with reverse transcription, in either one-step or two-step, and unusually small target sequences. Here we used synthetic oligonucleotides homologous to known miRNA, either in *C*. *elegans* or *H*. *sapiens*. We discovered that one issue with synthetic oligonucleotides is variation in production efficiency and purity. The quantitative UV absorbance measurement of our hsa-miR-155 synthetic oligonucleotide was artificially high, digital gel electrophoresis showed multiple bands, and digital capillary electrophoresis demonstrated a broad band with no clear majority peak. When using the synthetic hsa-miR-155 oligonucleotide for preliminary titration experiments, the experimental values were significantly diminished from the predicted values, especially in comparison to all other synthetic oligonucleotides. Upon further research we noted that these skewed measurements could be related to miRNA hsa-miR-155 containing a guanine quadruplex that makes it difficult to synthesize artificially [35]. A second batch of the synthetic hsa-miR-155 was produced using RNase free HPLC purification; initial evaluation of this new synthetic hsa-miR-155 oligonucleotide using chip-based automated electrophoresis system shows a tight peak and single band, both indicating a much cleaner product. We conclude that: (1) not all synthetic miRNA oligonucleotides are suitable for measurement standards and (2) there is a need to check purity of synthetic oligonucleotides using multiple modalities prior to proceeding with development of any assays.

Earlier publications focused on evaluating one-step versus two-step RNA quantification, different reagents used within the ddPCR or qRT-PCR quantification, and the use of endogenous controls versus spike-in controls [4, 6, 13, 14, 36]. Here, our sole interest is to determine how to leverage the current products on the market to make them more accurate, unbiased, and metrologically traceable [8] when quantitatively measuring biomarkers. We therefore employed very basic optimization techniques such as evaluating PCR annealing temperature. Table 5 shows the drastic number of non-specific amplification products that are observed with each of the kits. Since each kit uses different technologies for miRNA reverse transcription, one can hypothesize the possibility of different optimized annealing temperatures for each associated reverse transcription kit. It is therefore very important to validate annealing temperature with a few different template types prior to solidifying the experimental design.

It is very difficult to accurately measure the exact conversation rate associated with the reverse transcription in any cDNA synthesis reaction. All four kits demonstrated less than ideal conversion rate between miRNA and their end-step as measured via ddPCR. We argue that one should be able to formulate a model that allows accurate quantification of any miRNA in a sample via a series of normalization steps by simultaneously measuring at least three known and validated miRNAs in the same sample for the same reaction. Here we used two spike-in controls homologous to *C*. *elegans*. And while we tested some other *C*. *elegans* miRNA homologs, we found them to have more NTC and NEC positive droplets, significantly different annealing temperatures, or other sources of measurement difficulties. The use of non-cognate spike-in controls was also investigated by NIST scientists and we believe this option shows significant potential given the propensity for spike-in controls homologous to other organism to still hybridize to regions of human mRNA (S2 Fig). And while we accounted for this measurement bias when calculating uncertainty, these off-target effects still limit our assay range, specificity, and sensitivity. The only major obstacle with using non-cognate spike-in controls is the lack of readily available reagents for all the cDNA synthesis kits we tested. This option seems viable and favorable for future applications if it can be made cost-effective.

Spike-in controls also suffer from high between-sample variability [37], we therefore recommend each sample be measured in tandem with the spike-in controls and vetted endogenous markers (Fig 6C). Furthermore, the strength of the model is improved by increasing the number of spike-in controls to create “pools” of targets at a larger dynamic range [32]. There are limitations to increasing the diversity and dynamic range of spike-in controls, including economic constraints and amount of sample material. As we have shown here, by performing validated miRNA titration with just three targets, one can apply the power curve to create a new model that is used to compute exact miRNA measurements for subsequent measurements in that sample. It is important to note however, that with multiple freeze-thaws or dilutions, miRNA measurements can change. Thus, it is our additional recommendation to run a vetted endogenous marker and spike-in control for every sample and every experiment.

We originally choose to test two endogenous miRNAs to standardize and measure along with our spike-in controls. There is a potential for spike-in controls to suffer from different measurement challenges then endogenous measurements and therefore to have confidence in your model, we suggest that at least one endogenous marker be used. However, the same endogenous miRNAs should not be applied as standardized controls for all sample types and measurement since there is no proven consistently expressed miRNA for all samples [3]. We therefore recommend selecting endogenous miRNAs that are ubiquitously expressed at higher levels in the sample of interest to titrate and validate as “endogenous markers” for one’s specific assay. These markers should be calibrated similarly to the spike-in controls by creating titration curves that evaluate their random and independent dispersion among droplets, measurement linearity, and limit of quantification. In our initial screening and validation, we discovered that the synthetic oligonucleotide to hsa-miR-155 was inadequate for synthetic miRNA titration relative endogenous measurements. We therefore proceeded with hsa-miR-223 as our endogenous marker.

Synthetic miRNA oligonucleotide titration quantification and model fitting prior to running an assay on patients is important to understand limitations and normalizations for an assay. For instance, all the cDNA kits tested have characteristic limits of quantification (LOQ)—the minimum number of targets per droplet that can be confidently measured. Knowing the LOQ can help explain variability in experimental measurements if one dilutes the spike-in control or test sample too much (Fig 1A). Additionally, miRNA titration curves are needed to predict values in the test sample (Fig 7C, “Predicted”). This predicted value will ultimately lead to the model that applies to all unknown targets (Fig 1D). After this initial assay validation, one can rely on limited sample wells to test NTC, NEC, spike-in values, and endogenous markers to confirm the accuracy of an individual assay. If the individual sample assay is working properly, then plotting experimental versus predicted copy number with the endogenous marker will result in a highly correlated power model that can confidently be applied to all unknown markers on that plate (Fig 1E).

In addition to increasing the number of spike-in controls or endogenous markers, the predicted value of unknowns will be strengthened by running more biological and technical replicates. The next step for this project is to apply these quantitative measurement techniques to many normal human samples to determine normal physiological levels of miRNA of interest. Consensus and feasibility need to be addressed for each miRNA value to reduce uncertainty in the measurements and determine a clinically acceptable value.

In the current market of uncertainty around validity, safety, and effectiveness of bioassays used to benchmark disease diagnosis, prognosis, and treatment options, it is important to consider necessary improvements in robustness and accuracy of the measurement techniques. miRNA has a promising future in all areas of the biotechnology field, but it is important to first outline opportunities to standardize the tests. Here we propose a procedure that can be used to screen a single patient for abnormal miRNA levels. Leveraging digital PCR and whatever cDNA synthesis technology is preferred by the individual laboratory, these techniques can be applied rapidly to come up with accurate concentrations or copy numbers of miRNA in a high throughput manner.

## Supporting information

S1 Fig Titration steps for calibrating synthetic miRNA oligonucleotides. Example of dilutions performed to titrate synthetic miRNA oligonucleotides using a stock concentration of 100 μmol/L. Diagram shows microliter volume transferred between microcentrifuge tubes, dilutions, and corresponding concentration following dilution. Numbers (1)–(5) indicate concentrations that are small enough to detect on our droplet digital PCR instrument. Dilutions and concentrations are nominally defined.
(PDF)

S2 Fig Baseline cell-associated miRNA expression compared to spike-in values. Total RNA from additional THP-1 cells was isolated without the addition of synthetic miRNA spike-in, as described in the general methods section. Mean experimental lambda for cel-miR-238 (brown), cel-miR-39 (gray), hsa-miR-155 (white), and hsa-miR-223 (blue) is plotted for THP-1 cells without spiked-in synthetic miRNA (solid bars) and with spiked-in synthetic miRNA (checkered bars). Data from THP-1 cells with spiked-in synthetic miRNA is the same as plotted in Fig 7.
(PDF)

S3 Fig Quantification of cell-associated hsa-miR-155 in peripheral blood cell subsets. Total RNA was extracted from primary CD14+ monocytes and CD66b+CD16+ neutrophils. RNA was quantified and tested for integrity and then used to make cDNA using kit “B.” Targets of cel-miR-238, cel-miR-39, hsa-miR-223, and hsa-miR-155 per droplet were measured using droplet digital PCR. Cel-miR-238, cel-miR-39, and hsa-miR-223 targets per droplet were converted to copies per microliter using our titration curve described in Fig 6 and a power curve was generated for this sample. The sample-specific power curve was used to convert hsa-miR-155 targets per droplet into copies per microliter. Predicted has-miR-155 copy number with associated uncertainty is shown for each cell subset group.
(PDF)

S1 Methods Supplemental methods for supplemental figures. (PDF)

S1 Appendix Complete raw data files from this study. The complete raw data set can be accessed by clicking https://doi.org/10.18434/M32Q1V. A file titled “ddPCR Raw Data_Stein et al PLOSOne 2017.xlsx” will download.
(ZIP)
