# Supplementary material for: Steps to achieve quantitative measurements of microRNA using two step droplet digital PCR
Source: PLoS One. 2017 Nov 16;12(11):e0188085. doi: 10.1371/journal.pone.0188085 (PMC5690473; doi:10.1371/journal.pone.0188085)
Supplement: S1 Fig — Example of dilutions performed to titrate synthetic miRNA oligonucleotides using a stock concentration of 100 μmol/L. Diagram shows microliter volume transferred between microcentrifuge tubes, dilutions, and corresponding concentration following dilution. Numbers (1)–(5) indicate concentrations that are small enough to detect on our droplet digital PCR instrument. Dilutions and concentrations are nominally defined. (PDF) [file pone.0188085.s001.pdf]

Titration Steps for Calibrating Spike-In Controls

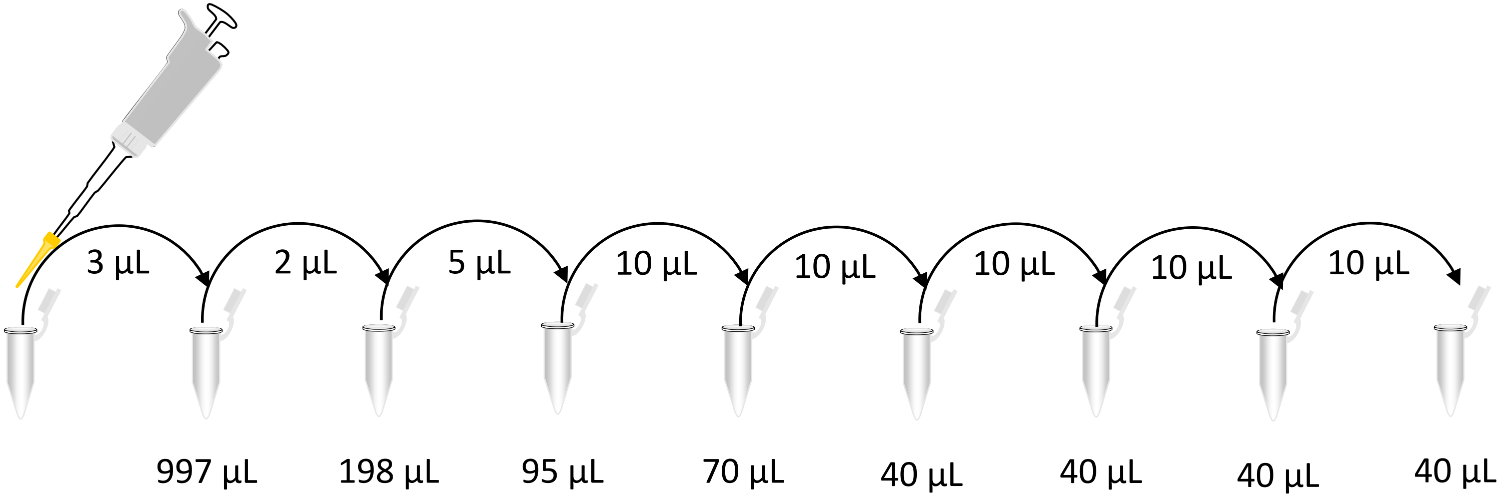

Dilution:	1.0×10^{-1}	3.0×10^{-4}	3.0×10^{-6}	1.5×10^{-7}	1.88×10^{-8}	3.75×10^{-9}	7.5×10^{-10}	1.5×10^{-10}	3.0×10^{-11}
Concentration:	$1 \times 10^{10} \frac{f\text{mole}}{\text{liter}}$	$3.0 \times 10^{7} \frac{f\text{mole}}{\text{liter}}$	$3.0 \times 10^{5} \frac{f\text{mole}}{\text{liter}}$	$1.5 \times 10^{4} \frac{f\text{mole}}{\text{liter}}$	$1.88 \times 10^{3} \frac{f\text{mole}}{\text{liter}}$	$3.75 \times 10^{2} \frac{f\text{mole}}{\text{liter}}$	$7.5 \times 10^{1} \frac{f\text{mole}}{\text{liter}}$	$1.5 \times 10^{1} \frac{f\text{mole}}{\text{liter}}$	$3.0 \times 10^{0} \frac{f\text{mole}}{\text{liter}}$
					(1)	(2)	(3)	(4)	(5)

S1 Fig. Titration Steps for Calibrating Synthetic miRNA Oligonucleotides. Example of dilutions performed to titrate synthetic miRNA oligonucleotides using a stock concentration of 100 µmol/L. Diagram shows microliter volume transferred between microcentrifuge tubes, dilutions, and corresponding concentration following dilution. Numbers (1) – (5) indicate concentrations that are small enough to detect on our droplet digital PCR instrument. Dilutions and concentrations are nominally defined.
