# Supplementary material for: Steps to achieve quantitative measurements of microRNA using two step droplet digital PCR
Source: PLoS One. 2017 Nov 16;12(11):e0188085. doi: 10.1371/journal.pone.0188085 (PMC5690473; doi:10.1371/journal.pone.0188085)
Supplement: S2 Fig — Total RNA from additional THP-1 cells was isolated without the addition of synthetic miRNA spike-in, as described in the general methods section. Mean experimental lambda for cel-miR-238 (brown), cel-miR-39 (gray), hsa-miR-155 (white), and hsa-miR-223 (blue) is plotted for THP-1 cells without spiked-in synthetic miRNA (solid bars) and with spiked-in synthetic miRNA (checkered bars). Data from THP-1 cells with spiked-in synthetic miRNA is the same as plotted in Fig 7. (PDF) [file pone.0188085.s002.pdf]

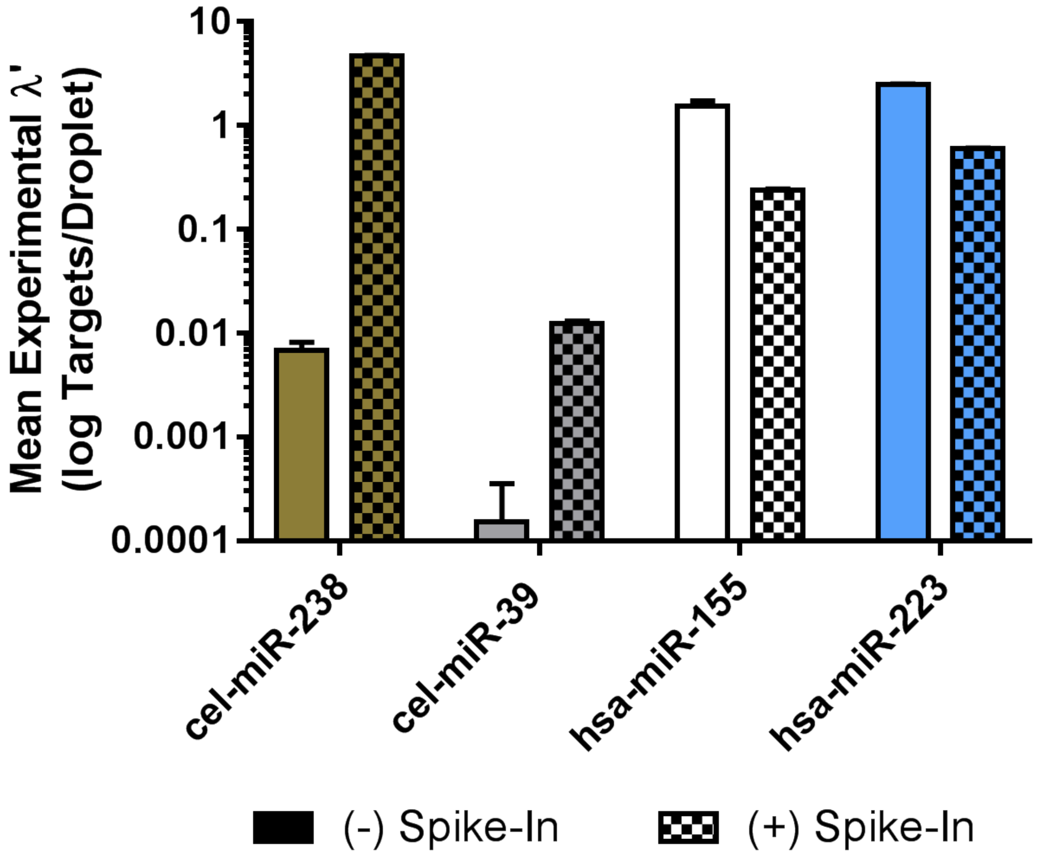

S2 Fig. Baseline cell-associated miRNA expression compared to spike-in values. Total RNA from additional THP-1 cells was isolated without the addition of synthetic miRNA spike-in, as described in the general methods section . Mean experimental lambda for cel-miR-238 (brown), cel-miR-39 (gray), hsa-miR-155 (white), and hsa-miR-223 (blue) is plotted for THP-1 cells without spiked-in synthetic miRNA (solid bars) and with spiked-in synthetic miRNA (checkered bars). Data from THP-1 cells with spiked-in synthetic miRNA is the same as plotted in figure 7.
