# Supplementary material for: Steps to achieve quantitative measurements of microRNA using two step droplet digital PCR
Source: PLoS One. 2017 Nov 16;12(11):e0188085. doi: 10.1371/journal.pone.0188085 (PMC5690473; doi:10.1371/journal.pone.0188085)
Supplement: S3 Fig — Total RNA was extracted from primary CD14+ monocytes and CD66b+CD16+ neutrophils. RNA was quantified and tested for integrity and then used to make cDNA using kit “B.” Targets of cel-miR-238, cel-miR-39, hsa-miR-223, and hsa-miR-155 per droplet were measured using droplet digital PCR. Cel-miR-238, cel-miR-39, and hsa-miR-223 targets per droplet were converted to copies per microliter using our titration curve described in Fig 6 and a power curve was generated for this sample. The sample-specific power curve was used to convert hsa-miR-155 targets per droplet into copies per microliter. Predicted has-miR-155 copy number with associated uncertainty is shown for each cell subset group. (PDF) [file pone.0188085.s003.pdf]

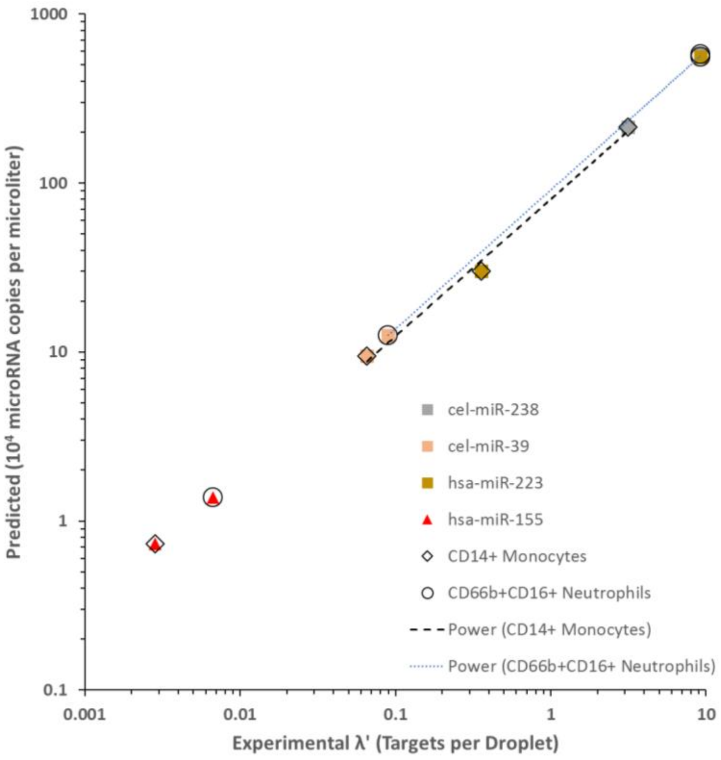

Cell Type	R^2	a	b	Predicted hsa-miR-155 Copy Number (10^4)
Monocyte (CD14+)	0.994	80.02	0.810	0.7329 ± 0.117
Neutrophils (CD66b+CD16+)	0.999	90.92	0.821	1.382 ± 0.038

S3 Fig. Quantification of cell-associated hsa-miR-155 in peripheral blood cell subsets. Total RNA was extracted from primary CD14+ monocytes and CD66b+CD16+ neutrophils. RNA was quantified and tested for integrity and then used to make cDNA using kit "B." Targets of cel-miR-238, cel-miR-39, hsa-miR-223, and hsa-miR-155 per droplet were measured using droplet digital PCR. Cel-miR-238, cel-miR-39, and hsa-miR-223 targets per droplet were converted to copies per microliter using our titration curve described in figure 6 and a power curve was generated for this sample. The sample-specific power curve was used to convert hsa-miR-155 targets per droplet into copies per microliter. Predicted has-miR-155 copy number with associated uncertainty is shown for each cell subset group.
