# Supplementary material for: Steps to achieve quantitative measurements of microRNA using two step droplet digital PCR
Source: PLoS One. 2017 Nov 16;12(11):e0188085. doi: 10.1371/journal.pone.0188085 (PMC5690473; doi:10.1371/journal.pone.0188085)
Supplement: S1 Methods — (PDF) [file pone.0188085.s004.pdf]

S1 Methods. Supplemental methods for supplemental figures.

Monocyte and Neutrophil Isolation

Anonymous whole blood samples in EDTA were obtained from NIH's Department of Transfusion Medicine according to their institutional review boards guidelines. Whole blood was mixed in equal dilution with Hank's Balanced Salt Solution, containing no calcium or magnesium (HBSS; MediaTech, Manassas, VA) and laid over Ficoll-Plaque PLUS (GE Healthcare, Pittsburg, PA). Peripheral blood mononuclear cells (PBMCs) were removed and blood layer was saved. Both blood layer and PBMC layer were washed with magnetic bead separation buffer (2 % fetal calf serum, 1 mmole/L ethylenediaminetetraacetic acid in phosphate buffered saline, pH 7.4 buffer). CD14+ monocytes were isolated from the PBMC layer using the EasySep Human Monocyte Isolation Kit (StemCell Technologies, Vancouver, BC) and CD66b+CD16+ neutrophils were isolated from the blood layer using the EasySep Human Neutrophil Enrichment Kit (StemCell Technologies, Vancouver, BC), both using manufacturer's instructions. Cells were counted using trypan blue and a hemocytometer to obtain a nominal value. An aliquot of cells was removed to verify phenotype using flow cytometry as described below and remaining cells were flash frozen in RNAse-free microcentrifuge tubes using liquid nitrogen.

Cell Phenotype Verification

Cells were washed with 2 % fetal calf serum, 1 mmole/L ethylenediaminetetraacetic acid in phosphate buffered saline, pH 7.4 buffer and antibodies to detect cell surface antigens were added. Monoclonal antibodies, anti-human CD16 phycoerythrin-cyanine 5 (PE/Cy5, clone 3G8; ThermoFisher Scientific, Grand Island, NY), anti-human CD66b pacific blue (clone G10F5; ThermoFisher Scientific, Grand Island, NY), anti-human CD45 fluorescein (FITC, clone HI30; BD Biosciences, San Diego, CA), anti-human CD4 brilliant violet 510 (BV510, clone SK3; ThermoFisher Scientific, Grand Island, NY), anti-human CD14 phycoerythrin (PE, clone 61D3; ThermoFisher Scientific, Grand Island, NY). Stained cells were analyzed on a FACSAria II (BD Biosciences, San Diego, CA) and flow cytometry data was analyzing using FlowJo (v10; Ashland, OR).

Table. Nominal Cell Counts for Each Sample

Cell Type	Contains Spike-In Controls	Cell Counts Per Extraction
THP-1 Cells	Yes	1.9×10^6
THP-1 Cells	No	6.97×10^6
CD14+ Monocytes	Yes	1.5×10^5
CD66b+CD16+ Neutrophils	Yes	1.0×10^7
